# MicroRNA-939 inhibits cell proliferation via targeting LRSAM1 in Hirschsprung’s disease

**DOI:** 10.18632/aging.101331

**Published:** 2017-12-18

**Authors:** Guanglin Chen, Chunxia Du, Ziyang Shen, Lei Peng, Hua Xie, Rujin Zang, Hongxing Li, Yankai Xia, Weibing Tang

**Affiliations:** 1 State Key Laboratory of Reproductive Medicine, Institute of Toxicology, School of Public Health, Nanjing Medical University, Nanjing 211166, China; 2 Key Laboratory of Modern Toxicology (Nanjing Medical University), Ministry of Education, China; 3 Department of Pediatric Surgery, Children’s Hospital of Nanjing Medical University, Nanjing 210008, China; 4 Department of Endocrinology and Metabolism, The First Affiliated Hospital of Nanjing Medical University, Nanjing, China; * Equal contribution

**Keywords:** Hirschsprung's disease, microRNA (miRNA), proliferation, migration, autophagy

## Abstract

**Background:**

Hirschsprung's disease (HSCR) is a common digestive disease caused by impaired development of neural crest cells. Some studies have revealed the roles of microRNA (miRNA) in various diseases. But the function of miRNA in HSCR needs further investigation.

**Methods:**

We adopted qRT-PCR and immunoblot analyses to explore the relative expression of miR-939 and LRSAM1 in 80 HSCR bowel tissues and 80 normal bowel tissues. CCK-8 assay, transwell assay and flow cytometry were used to evaluate the function of miR-939 by overexpression of miR-939 in 293T, SK-N-BE(2), SH-SY5Y cell lines. The direct connection between miR-939 and LRSAM1 was validated by dual-luciferase reporter assay. We also investigated the autophagy level via immunoblot analyses.

**Results:**

Mir-939 was significantly upregulated in HSCR tissues with decreased expression of LRSAM1. Overexpression of miR-939 suppressed cell proliferation without affecting cell apoptosis, cell cycle or cell migration. And LRSAM1 exerted similar function. Autophagy was impaired in HSCR tissues compared with control samples. Mir-939 did not inhibit the autophagy although it decreased the expression of LRSAM1.

**Conclusions:**

Our study shows the potential function of mir-939 through regulating LRSAM1 in HSCR and infers that autophagy may also confer the risk of HSCR.

## Introduction

Hirschsprung's disease (HSCR) characterized by the absence of intestinal ganglion cells in myenteric and submucosal plexuses is a developmental malformation of enteric nervous system which affects 1:5000 live births and has a male preponderance of 4:1 [1,2]. HSCR is a complex disease caused by multiple factors including genetic and environmental factors that regulate enteric neural crest cells (ENCCs) proliferation, differentiation and migration in the embryonic stage [3,4].

MicroRNAs (miRNAs) are endogenous small non-coding RNAs ranging in size from 19 to 25 nucleotides and play important roles in multiple cellular process such as proliferation, apoptosis, development, and differentiation [5,6]. They mainly regulate gene expression by targeting to the complementary mRNA and resulting in mRNA cleavage and/or translation repression [7]. Dysregulation of miRNAs has been shown to be associated with HSCR such as miR-206, miR-192/215 by suppressed cell proliferation [8,9]. Our previous study through miRNA microarray has found numerous dysregulated miRNA in HSCR samples. MiR-939 was significantly upregulated in HSCR cases compared with matched controls (data not shown). Recent studies have demonstrated that miR-939 is responsible for ovarian cancer due to its impact on cell proliferation and regulates human inducible nitric oxide synthase gene expression [10,11]. However, the role of miR-939 in the pathological process of HSCR has not been reported until now.

Autophagy is a highly regulated biological mechanism involved in recycling cellular constituents and intracellular degradation of damaged organelles and protein aggregates [12,13]. In additional to housekeeping and homeostatic functions at basal level, autophagy is initiated under stress conditions such as nutrient or growth factor deprivation, hypoxia and inflammation [14-16]. Autophagy has participated in various diseases including cancer and neurodegenerative disorders [17,18]. According to available miRNA databases, LRSAM1 is a potential target of miR-939, which is associated with autophagy induction and motor neuropathies. However, whether LRSAM1 participates in the development of HSCR remains to be addressed.

In this study, we demonstrate that how miR-939 interacts with its target gene LRSAM1 and explore the autophagy level in HSCR.

## RESULTS

### Clinical specimens

The Table 1 summarized the clinical information obtained from all participants including age, gender (male/female) and body weight. There were no statistically significant differences in terms of age and body weight. The male/female ratio of HSCR cases was 3.7:1, which was consistent with the gender rate of this disease. In total, 80 HSCR cases and 80 matched controls were enrolled in this study.

**Table 1:** Demographic clinical features of study subjects

**Variable**	**Control(n=80)**	**HSCR(n=80)**	**P**
Age(days,mean,SE)	127.91(7.65)	115.42(6.67)	0.22
Weight(kg,mean,SE)	5.53(0.15)	5.32(0.13)	0.30
Sex(%)			
Male	56(70.00)	63(78.75)	0.11
Female	24(30.00)	17(21.25)	

### Overexpression of miR-939 in HSCR

**Figure 1:**
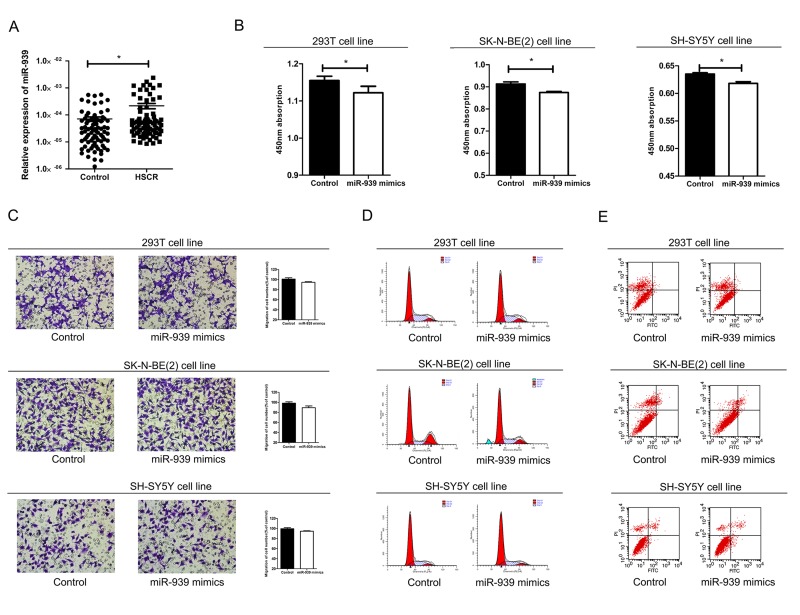
**Mir-939 was upregulated in HSCR tissues and cytobiology change after treating cells with its mimics.** (**A**) Mir-939 was significantly overexpressed in HSCR (n=80) tissues compared with control samples (n=80). Human 293T, SK-N-BE(2), SH-SY5Y cell lines were transfected with miR-939 mimics, upregulated mir-939 suppressed cell proliferation indicated by the CCK-8 assay (**B**) without impact on cell migration (**C**), cell cycle (**D**) and cell apoptosis (**E**). *indicates significant difference compared with control group, P<0.05.

qRT-PCR was used to examine the expression level of miR-939 in all colon tissues, and showed that the expression level of miR-939 was significantly higher in HSCR cases compared with matched controls (Fig. 1A). Similar results could be found in ganglionic and aganglionic intestine of the same HSCR participants (Supplemental Fig. 1A). The results indicated that miR-939 might play an important role in the pathological development of HSCR.

### MiR-939 mimics decreased cell proliferation

In order to confirm the effect of miR-939 on cell function in vitro, cell migration, cell proliferation, cell cycle and apoptosis were examined by transfecting the 293T, SH-SY5Y and SK-N-BE(2) cell lines with miR-939 mimics. Overexpression of miR-939 significantly reduced the number of proliferating cells (Fig. 1B). Flow cytometry analysis was conducted to investigate the role of miR-939 in cell cycle and apoptosis. However, no statistical differences in the percentage of migrating cells, apoptotic cells or cell cycle process were detected between cells treated with miR-939 mimics and the negative control (Fig. 1C-E).

### Target prediction of miR-939

To investigate the molecular mechanism by which miR-939 affects cell function, we used three miRNA databases (Targetscan, DIANA LAB and miRDB) to predict the target genes. After the prediction and functional analysis, three potential target genes LRSAM1, LNX2 and UBE2D3 were selected. LRSAM1 plays key roles in autophagy induction and is involved in motor neuropathies [19,20]. LNX2 is a member of LNX family expressed in nervous system and promotes neuronal cell proliferation and differentiation [21,22]. UBE2D3 is a member of the E2 family and regulates basic cellular activities including cell cycle, DNA damage response and apoptosis [23].

### LRSAM1 was down-regulated in HSCR tissues

**Figure 2:**
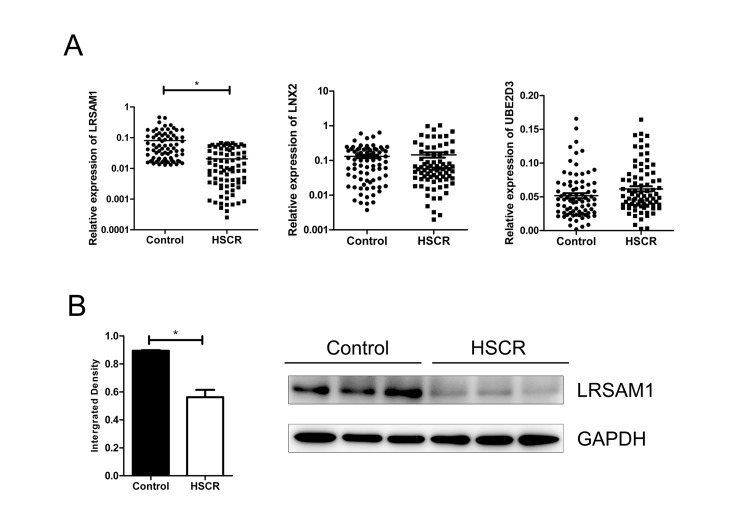
**LRSAM1 was down-regulated in HSCR samples.** (**A**) The expression of LRSAM1 significantly decreased in HSCR (n=80) tissues compared with control samples (n=80) in mRNA level. No significant difference of LNX2 or UBE2D3 was observed between HSCR and control. (**B**) The protein expression level of LRSAM1 in HSCR tissues and controls. *indicates significant difference compared with control group, P<0.05.

qRT-PCR was adopted to analyze the mRNA level of all the three predicted targeted genes in 80 cases of HSCR and 80 matched controls. LRSAM1 was the only candidate gene which was down-regulated in cases compared with controls. However, LNX2 and UBE2D3 showed no difference in expression level between cases and controls (Fig. 2A). Furthermore, the protein expression level of LRSAM1 was consistent with mRNA levels by western blot (Fig. 2B). Decreased expression of LRSAM1 could also be found in stenotic segment and matched dilated segment of the same HSCR cases (Supplemental Fig. 1B, C).

### LRSAM1 was target gene for miR-939

**Figure 3:**
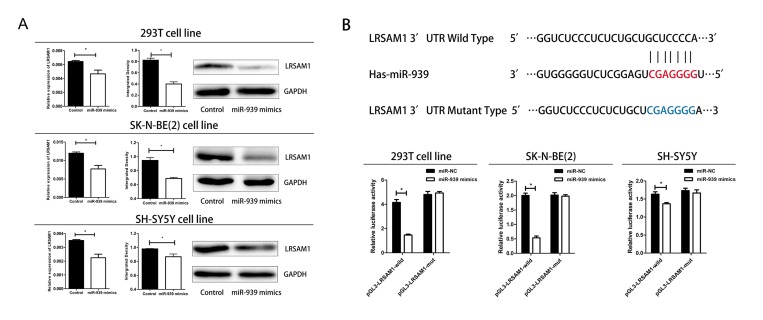
**Mir-939 directly regulates LRSAM1.** (**A**) Sequence alignment of human miR-939 with 3’UTR of LRSAM1. Bottom: mutations in the 3’-UTR of LRSAM1. (**B**) All cells were transfected with miR-939 and negative control, renilla luciferase vector pRL-SV40 and LRSAM13’UTR luciferase reporters. Both firefly and Renilla luciferase activities are measured in the same sample. Firefly luciferase signals were normalized with Renilla luciferase signals. *indicates significant difference compared with control group, P<0.05.

To confirm the miRNA-target interactions, 293T, SH-SY5Y and SK-N-BE(2) cell lines were transfected with miR-939 mimics. The results showed ectopic expression miR-939 significantly suppressed the mRNA and protein expression of LRSAM1 in three cell lines (Fig. 3A). In addition, miR-939 decreased the luciferase activity of the wild-type pGL3-LRSAM1 but not the mutant reporter gene GL3-LRSAM1-mut, indicating that miR-939 suppressed LRSAM1 by binding to the 3’UTR region (Fig. 3B). Next, we wondered whether suppression of LRSAM1 expression by siRNA has a similar function like miR-939. As shown in Supplemental Fig. 2, suppression of LRSAM1 decreased cell proliferation. The results suggested that miR-939 suppressed cell proliferation directly by regulating LRSAM1.

### Autophagy was suppressed in HSCR

**Figure 4:**
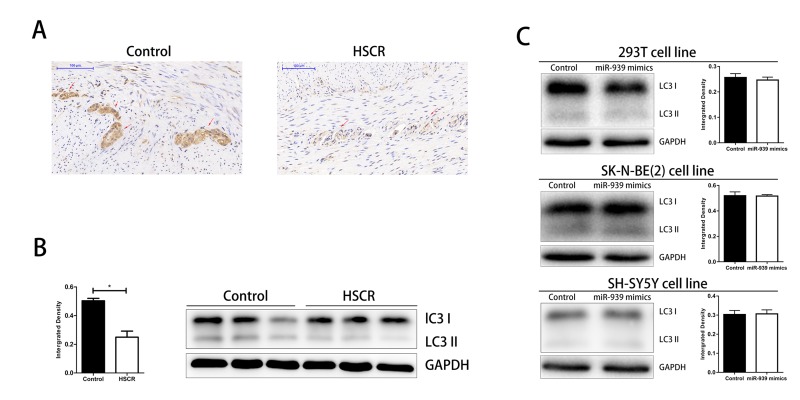
**Downregulated level of autophagy in HSCR.** The autophagy was significantly suppressed in HSCR via detecting the expression of LC3 through IHC (**A**) and WB (**B**). (**C**) Autophagy level was not changed when mir-939 was upregulated by mimics. *indicates significant difference compared with control group, P<0.05.

Given that LRSAM1 was an important gene involved in autophagy, we investigated the autophagy level in HSCR [20]. We detected the conversion of LC3-I to LC3-II by western bolt and immunohistochemical staining. As shown in Fig. 4 A-B, the level of autophagy was significantly lower in HSCR cases. We then wondered if miR-939 inhibited the autophagy in vitro. However, we found no difference between groups of miR-939 mimics and control ones. Results above hinted that autophagy was impaired in HSCR and miR-939 did not participate in this biological process.

## DISCUSSION

HSCR is one of the most common digestive diseases with the symptoms of constipation, vomiting and abdominal distension in the new born. Nowadays, the main treatment of HSCR is to remove the aganglionic bowels. However, the long term outcome is not always satisfactory especially when patients with enterocolitis [24,25]. The cost for intinial hospitalization and time of hospital stay is $100,000 and a month on average respectively [26]. Therefore, it is essential to elucidate the exact pathogensis underlying HSCR which can offer a better understanding in treatment and early diagnosis.

In our study, we found potential targeted genes of miR-939 using Targetscan, DIANA LAB and miRDB. LRSAM1 was significantly differentially expressed between HSCR cases and matched controls. Then, we used dual-luciferase report assay to validate that LRSAM1 is directly regulated by miR-939. In vitro results showed that up-regulation of miR-939 suppressed cell proliferation without influencing cell migration, cell cycle and cell apoptosis. Meanwhile, the similar results generated by LRSAM1 siRNA hinted that miR-939 performed its function by regulating LRSAM1.

LRSAM1 (leucine-rich repeat and sterile alpha motif-containing protein 1) is a highly conserved ubiquitin-protein ligase, which is expressed in the peripheral neural system as well as the central neuron system [27,28]. Previous studies showed that mutation of LRSAM1 is associated with neurological disorders such as Charcot-Marie-Tooth and Huntington’s disease [19,29]. Meanwhile, LRSAM1 also participates in WNT signaling which is related to HSCR [30]. Thus, we speculate that miR-939 could contribute to the pathogenesis of HSCR through negative regulation of LRSAM1. Moreover, LRSAM1 has been taken as important regulators of autophagy against bacteria via its LRR domain and RING domain [31]. Therefore, we detect the autophagy level in HSCR and the result shows higher level in matched controls. But LC3 II/LC3 I do not show significant difference between miR-939 mimics and control ones according to in vitro results. It is well known that one miRNA always has not only one targeted gene. So miR-939 may also affect other genes related to autophagy. But further research is needed to explore the meaningless effect of miR-939 on autophagy.

In conclusion, our data reveal that miR-939 is related to pathogenesis of HSCR via regulation of LRSAM1 and autophagy level is impaired in HSCR. Our research may shed new light in understanding the relationship between HSCR and autophagy.

## MATERIALS AND METHODS

### Tissues collection

All study subjects including 80 HSCR case samples and 80 matched controls were derived from patients undergoing surgical procedures at Nanjing Children’s Hospital Affiliated to Nanjing Medical University from October 2009 to May 2014. The diagnosis of HSCR was confirmed by pathological analysis after surgery. The matched controls were collected from patients’ intestinal without the ischemia or necrosis parts, which were proven to be without HSCR or other congenital malformations. All tissue samples were stored at -80 ^o^C immediately after surgery. Written informed consent was obtained from patients’ guardians according to the legal institutional guidelines and ethical permission.

### QRT-PCR

Total RNA, including miRNA, was extracted using Trizol reagent (Life technologies, CA, US). For microRNA analysis, we used TaqMan® MicroRNA Assays (Applied Biosystems, CA, US) as the probe for hsa-miR-939 and hsa-U6 which served as an internal control. For the mRNA detection, the mRNA was measured by ABI 7900HT along with the GAPDH as the endogenous control. Primer sequences were listed in Supplemental Table 1.

### Antibodies and Western blot (WB)

The tissue samples and cells were lysed using a RIPA buffer (Beyotime, Nantong, China). The membranes with the target protein were incubated with the primary antibody at 4 ^o^C overnight and then incubated with the secondary antibody (Beyotime, Nantong, China) for 1 hour at room temperature. The primary antibodies used were LC3A/B (12741, cell signaling), GAPDH (sc-25778, Santa Cruz) and LRSAM1(ab171556, abcam). Image J software was used to detect and quantify the protein level in the Western Blot.

### Cell culture and transfection

The 293T, SK-N-BE(2) and SH-SY5Y cell lines were obtained from American Type Culture Collection (ATCC, Manassas VA, USA) and cultured in DMEM medium (Hyclone, UT, US) supplemented with 10% heat-inactivated fetal bovine serum (FBS), penicillin (100U/ml) and streptomycin (100U/ml) under the condition of 37 ^o^C and 5% CO_2_. The miRNA precursor molecules of miR-939, siRNA of LRSAM1 and negative control (GenePharma, Shanghai, China) were used in transfection experiments with Lipofectamine 2000 Reagent (Invitrogen,CA, US) according to manufacturer’s instructions.

### Cell transwell assays

Transwell migration chambers (8 um pore size, Millipore Corporation, Billerica, MA) were used to evaluate the capacity of cell migration after transfecion of cells with miR-939 mimics and negative control. About 100ul cell suspension with serum-free medium were seeded in the upper chamber (1×10^6^ cells/ml) with the 600ul medium containing 10% fetal bovine serum in the lower chamber. After 48h transfection, cells were stained with crystal violet staining solution (Beyotime, Nantong, China), counted and photographed under 40 × magnification (five views per well). The number of migrated cells was counted using Iamge-pro Plus 6.0 while the amout of control cells was normalized to 1.

### Cell proliferation assays

CCK-8 assay (Beyotime, Nantong, China) was conducted to evaluate the cell proliferation after 24h transfection. We used TECAN infinite M200 Multimode microplate reader (Tecan, Mechelen, Belgium) to measure the absorbance at 450nm.

### Cell cycle and apoptosis analysis

For the cell cycle assay, cells transfected with miR-939 mimics were collected and detected by BD Biasciences FACS Calibur Flow Cytometry (BD Biasciences, NJ, US). For detection of apoptosis, cells were harvested and stained with the Annexin V-FITC/Propidium Iodide Kit (KeyGen Biotech, Nanjing, China) according to the manufacturer’s instructions. Data were analyzed with FlowJo V7 software (Tree Star, Ashland, OR, US).

### Dual-luciferase reporter assay

The wild-type and mutated 3’-UTR sequence of LRSAM1 mRNA named pGL3-LRSAM1 and pGL3-LRSAM1-mut respectively were inserted into the KnpI and SacI sites of pGL3 promoter vector (Genescript, Nanjing, China). After transfection with negative control, miR-939 mimics, pGL3-LRSAM1 and pGL3-LRSAM1-mut for 48h following the manufacturer’s protocols, cells were collected and measured using the Dual Luciferase Assay (Promega, Madison, WI).

### Immunohistochemical staining (IHC)

For immunohistochemistry, sections of fixed and paraffin-embedded tissue samples were processed in Pathology Department of Nanjing Children’s Hospital. After deparaffinization and hydration, the sections were autoclaved in 0.01M trisodium citrate buffer for antigen retrieval and blocked by normal goat serum. Then specimens were incubated with anti-LC3A/B (12741, cell signaling, 1:2000 dilution) overnight at 4 ^o^C, and then stained using biotinylated goat anti-rabbit IgG secondary for 1h, followed by incubating with 3,3’-diaminobenzidine (Beyotime, Nantong, China). Intensity of staining was graded as follows: negative(-); low staining(+); intensive staining(++).

### Statistical analysis

Data obtained from tissue samples are analyzed using Wilcoxon rank-sum test while the the data of cell samples are presented as mean+SEM by double-sided Student’s t-test. All statistical analysis were performed using STATA9.2 (StataCorp, College Station, TX, US), and a p value of <0.05 was considered to be statistically significant. Qualitative data were representative of three independent experiments, with each performed in triplicate.

## Supplementary Material

Supplementary File
