# Correction: MicroRNA-939 inhibits cell proliferation via targeting LRSAM1 in Hirschsprung’s disease

**DOI:** 10.18632/aging.101650

**Published:** 2018-11-12

**Authors:** Guanglin Chen, Chunxia Du, Ziyang Shen, Lei Peng, Hua Xie, Rujin Zang, Hongxing Li, Yankai Xia, Weibing Tang

**Affiliations:** 1 State Key Laboratory of Reproductive Medicine, Institute of Toxicology, School of Public Health, Nanjing Medical University, Nanjing 211166, China; 2 Key Laboratory of Modern Toxicology (Nanjing Medical University), Ministry of Education, China; 3 Department of Pediatric Surgery, Children’s Hospital of Nanjing Medical University, Nanjing 210008, China; 4 Department of Endocrinology and Metabolism, The First Affiliated Hospital of Nanjing Medical University, Nanjing, China; * Equal contribution

Original article: Aging (Albany NY) 2017; 9: 2471-2479

**This article has been corrected:** The authors have submitted the wrong composite of Figure 1 (C), the mistake was in the 293T and SH-SY5Y cell line. The corrected panel C of Figure 1 is provided below. The authors declare that this correction does not change the results or conclusions of this paper. The authors sincerely apologize for this error.

**Figure 1:**
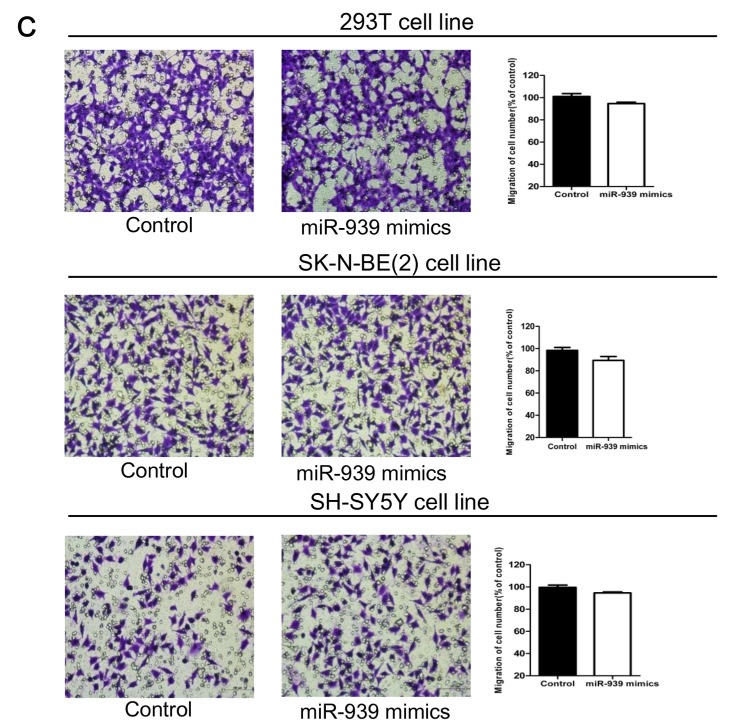
**Mir-939 was upregulated in HSCR tissues and cytobiology change after treating cells with its mimics.** (**A**) Mir-939 was significantly overexpressed in HSCR (n=80) tissues compared with control samples (n=80). Human 293T, SK-N-BE(2), SH-SY5Y cell lines were transfected with miR-939 mimics, upregulated mir-939 suppressed cell proliferation indicated by the CCK-8 assay (**B**) without impact on cell migration (**C**), cell cycle (**D**) and cell apoptosis (**E**). *indicates significant difference compared with control group, P<0.05.

