# Supplementary material for: MicroRNA-939 inhibits cell proliferation via targeting LRSAM1 in Hirschsprung’s disease
Source: Aging (Albany NY). 2017 Dec 18;9(12):2471–9. doi: 10.18632/aging.101331 (PMC5764386; doi:10.18632/aging.101331)
Supplement: Supplementary File [file aging-9-101331-s001.pdf]

SUPPLEMENTARY MATERIAL

Table S1. Primer sequence used for qRT-PCR and miR-939, siRNA related sequence.

LRSAM1	F: AAGGTGCTGATCGTCCACAC
	R: TGCCCCAGATCGTCAGGAA
UBE2D3	F: CCATATCAAGGCGGTGTATTCTT
	R: GGCGACCACTGTGATCTTAGA
LNX2	F: GGAACAACAAGTGATGAGATGGT
	R: CACAGGGTGTGTCTAGTGGC
GAPDH	F: GCACCGTCAAGGCTGAGAAC
	R: GGATCTCGCTCCTGGAAGATG
LRSAM1 siRNA	Sense: GCUCUCAGAGAUUCCAUUUTT
	Antisense: AAAUGGAAUCUCUGAGAGCTT
Negative control	Sense: UUCUCCGAACGUGUCACGUTT
	Antisense: ACGUGACACGUUCGGAGAATT
Hsa-miR-939 mimics	Sense: UGGGGAGCUGAGGCUCUGGGGGUG
	Antisense: CCCCCAGAGCCUCAGCUCCCCAUU
Negative control	Sense: UUCUCCGAACGUGUCACGUTT
	Antisense: ACGUGACACGUUCGGAGAATT

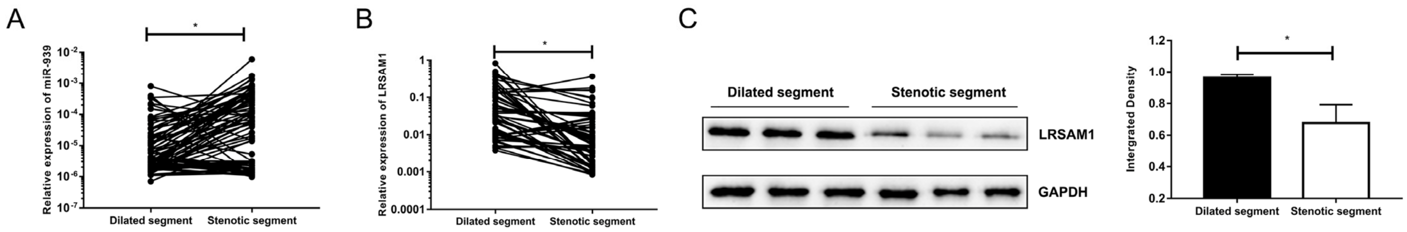

Figure S1. Expression of miR-939 and LRSAM1 in different segments of HSCR bowels. (**A**) Mir-939 was significantly overexpressed in stenotic segment compared with dilated segment of HSCR (n=80). (**B**, **C**) Expression of LRSAM1 significantly increased in dilated segment of HSCR in mRNA (**B**) and protein (**C**) (n=80). *indicates significant difference compared with control group, P<0.05.

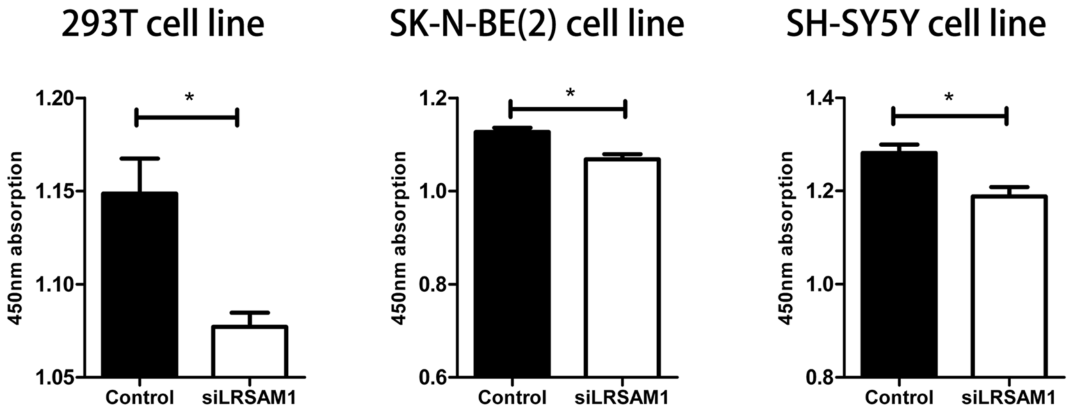

Figure S2. Knockdown of LRSAM1 suppressed cell proliferation. All cells transfected with LRSAM1 siRNA showed impaired cell proliferation compared with control. *indicates significant difference compared with control group, P<0.05.
